# Health literacy and pain neuroscience education in an interdisciplinary pain management programme: a qualitative study of patient perspectives

**DOI:** 10.1097/PR9.0000000000001093

**Published:** 2023-10-18

**Authors:** Janke Oosterhaven, Christopher D. Pell, Carin D. Schröder, Hans Popma, Loes Spierenburg, Walter L.J.M. Devillé, Harriet Wittink

**Affiliations:** a Research Group Lifestyle and Health, University of Applied Sciences Utrecht, Utrecht, The Netherlands; b Amsterdam Institute for Social Science Research, University of Amsterdam, Amsterdam, the Netherlands; c Department of Global Health, Academic Medical Centre, Amsterdam Institute for Global Health and Development (AIGHD), University of Amsterdam, Amsterdam, the Netherlands; d Ecare4you, Amersfoort, the Netherlands; e Center of Excellence for Rehabilitation Medicine Utrecht, UMC Utrecht Brain Center, University Medical Center Utrecht, and de Hoogstraat Rehabilitation, Utrecht, the Netherlands; f Rehabilitation Centre Heliomare, Wijk aan Zee, the Netherlands; g Julius Centre for Health Sciences and Primary Care, University Medical Centre, Utrecht, the Netherlands

**Keywords:** Health literacy, Patient education, Qualitative research, Interview, Pain management, Musculoskeletal pain

## Abstract

**Introduction:**

Pain neuroscience education is part of interdisciplinary pain management programs (IPMPs). To date, the role of health literacy on patients' understanding of pain neuroscience education has not sufficiently been examined.

**Objectives:**

Drawing on interviews with patients with diverse levels of health literacy, this article explores patient perspectives on pain neuroscience education.

**Methods:**

Purposively sampled patients from an IPMP were interviewed twice (waiting list and after 4 weeks). A directed qualitative content analysis was performed with the Integrated Conceptual Model of Health Literacy as an analytic framework.

**Results:**

Thirteen patients with chronic musculoskeletal pain were interviewed: 4 men and 9 women aged from 21 to 77 years with diverse educational and mostly low health literacy. One participant dropped out after baseline. Some participants gained access to health information actively; others relied on the expertise of their healthcare providers. Most participants did not seem to receive the information in the pain neuroscience education as intended, experienced difficulties with understanding the message, negatively appraised the information, and were not able to apply this in their daily lives. Health literacy levels likely played a role in this.

**Conclusions:**

Pain neuroscience education tailored to patients' health literacy levels, information needs, and learning strategies is needed.

## Abstract 2

Supplemental Digital Content is Available in the Text.

This qualitative study explores perspectives on pain neuroscience education by patients with various levels of health literacy in an interdisciplinary pain management program.

## 1. Introduction

Patients with persistent chronic musculoskeletal pain are frequently referred to interdisciplinary pain management programmes (IPMPs). A biopsychosocial approach, based on the understanding that chronic pain should be viewed as a dynamic interaction involving biological, psychological, and social factors unique to each individual, is used to help patients develop effective pain management strategies.^9,41^ Pain neuroscience education (PNE), an evidence-based form of pain education, aims to reconceptualize patients' pain, encouraging them to understand it through a biopsychosocial (rather than biomedical) lens by explaining pain biology.^5,10,15,23^ A systematic review has shown that PNE reduces catastrophising and increases knowledge about the complexity of pain.^10^ Pain neuroscience education is therefore an important part of these programs to improve patients' understanding of their chronic musculoskeletal pain. Pain neuroscience education outcomes are expected to be highly dependent on whether the patient can grasp the content of the education. Low levels of health literacy, defined as the ability to obtain, understand, and use health information to make important decisions regarding health and medical care,^18^ are associated with limited health-related knowledge and comprehension, a limited ability to deal with chronic disease and poor health outcomes.^1,19,32^ In patients with chronic pain, low health literacy has been associated with poorer pain-related knowledge, poorer self-management behaviours, including less adherence to medication regimens, greater pain intensity, greater kinesiophobia, and greater pain disability.^2,14,21^ Further research is needed to explore the role of health literacy on patients' understanding of PNE in IPMPs.

Drawing on a series of interviews with patients with diverse levels of health literacy enrolled in an IPMP, this article explores perspectives on PNE among patients with mostly low levels of health literacy. The Integrated Conceptual Model of Health Literacy was used to analyze the ways in which patients access, understand, appraise, and apply the health information in PNE.^32^ This model integrates the 3 domains of health care, disease prevention, and health promotion. As such, the model integrates the “medical” conceptualization of health literacy with the broader “public health” perspective. The core of the model shows the 4 types of competencies related to the process of accessing, understanding, appraising, and applying health-related information: (1) Access refers to the ability to seek, find, and obtain health information; (2) understand refers to the ability to comprehend the health information that is accessed; (3) appraise describes the ability to interpret, filter, judge, and evaluate the health information that has been accessed; and (4) apply refers to the ability to communicate and use the information to make a decision to maintain and improve health.

## 2. Materials and Methods

### 2.1. Setting and intervention

Data were collected as part of a larger study performed in the rehabilitation center Wijk aan Zee in the Netherlands. The outpatient IPMP (16–20 weeks, see Appendix 1, available as supplemental digital content at http://links.lww.com/PR9/A203) was based on cognitive-behavioral therapy including exercise therapy with PNE using a biopsychosocial explanation of chronic musculoskeletal pain. The focus of this study was the PNE in treatment phase 1 of the IPMP. The PNE was based on the “Explain Pain” manual as described by Butler et al.^3,23^ The average PNE group was about 4 to maximal 9 patients.

All group programme sessions of the specific disciplines of the IMPT team (for instance, physiotherapy or social worker) addressed the principles of PNE, but not in a structured organized format. In addition to these group sessions, all participants in the IPMP received a 2-hour group-based structured education session delivered by a physiotherapist and a psychologist. The education session was in the form of an interactive lecture; the education material was presented in a Microsoft PowerPoint presentation, and all participants were able to read along with the handouts. The participants were encouraged to ask questions, and a group discussion was held based on the educational material. The education was focused on the explanation of the pain system, the biological processes underpinning pain, the concept of central sensitization, and the influence of psychosocial issues in the individual pain experience.

### 2.2. Design

A series of semistructured interviews were conducted at 3 time points during the IPMP: waiting list (T0), after 4 weeks (T1), and at the end of the IPMP (T2). This article focuses on the semistructured interviews conducted at 2 time points: waiting list (T0) and after 4 weeks (T1), to examine patient perspectives on the pain education provided in the individual sessions and the group PNE sessions.

### 2.3. Participants and recruitment

Participants with chronic musculoskeletal pain attending the outpatient IPMP were recruited between April 2013 and December 2014. Chronic musculoskeletal pain was defined as pain persisting longer than 3 months or pain that extends beyond the expected period of healing.^22^ The rehabilitation team informed all patients on the waiting list about the study. Inclusion criteria for enrolment in the study were (1) patients with chronic musculoskeletal “noncancer pain,” (2) selected for treatment in the IPMP by the rehabilitation team, and (3) Dutch language competency. No relationship with the researchers was established before study commencement.

Purposive sampling was used to select male and female participants with diverse age and education levels; low (0–2: early childhood, primary education, lower secondary education), intermediate (3–5: upper secondary, postsecondary, short cycle tertiary), and high (6–8: bachelor, master's, doctoral^36^). The Short Assessment of Health Literacy–Dutch (SAHL-D)^26^ was used to ensure that participants with diverse health literacy levels were selected for this research study. The SAHL-D was the only valid objective measure of functional health literacy that was available in Dutch. The SAHL-D contains 33 items consisting of single words that refer to medical specialties, tests, treatments, and symptoms.^26^ Subjects have to pronounce each word of the test that has to be rated by a coder as either correct or incorrect. In addition, people have to select the correct meaning of each word using a multiple-choice format with 1 correct response option, 2 distractor options, and an “I don't know” option. One point is given for the correct meaning (comprehension test), and 1 point is given for the correct pronunciation (recognition test). Consequently, functional health literacy scores range from 0 to 66. Cronbach's alpha for comprehension is 0.79 and for recognition is 0.77.^26^

Functional health literacy is defined as the basic reading and writing skills needed to understand health information.^24^ The functional health literacy levels of the participants were reported in raw scores and described as either low or adequate based on the cutoff value of 54.5.^26^

Treatment fidelity measured as programme completion status was determined from registries by 2 independent researchers (J.O. and J.D.), and patients were classified as either a programme completer or noncompleter. Noncompleters were those patients with chronic pain, who were referred to the IPMP, who initiated (participated in the baseline assessments), but discontinued before completion of the entire programme.^25^

Data were collected until saturation was reached, ie, no new information emerged from the interviews.

### 2.4. Procedures

After pilot testing, semistructured interviews were conducted with patients who agreed to participate. Three female researchers (J.S., L.S., and J.O.) interviewed patients in pairs of 2 in their homes to ensure privacy. One researcher was an MSc physiotherapist and a PhD student, with an interest in patient dropout (J.O.), one an MSc anthropologist (J.S.), and one an MSc psychotherapist (L.S.). No others were present. The concept of health literacy^32^ guided the research questions, topic guide, and analysis process. The interview guide included open-ended questions on (1) the need for health information, (2) sources of health information, (3) use of health information (access, understand, appraise, and apply). Interviews lasted 1 to 1.5 hours. All interviews were digitally recorded and transcribed verbatim. Reflection notes were taken before and after the interviews.

### 2.5. Analysis

Analysis started alongside data collection to ensure that rich contextualized data were collected. The first 3 interviews were coded line by line for the following reasons: to reflect on the interview process, on the role of the researchers, to check whether the data adequately addressed the research questions, and to check whether new themes emerged from the data that should be addressed in the interview process and the analytical process.

Subsequently, the coding team (H.P., L.S., and J.O.) used the Integrated Conceptual Model of Health Literacy as an analytic framework for directed qualitative content analysis^32,33^ in Max Weber Qualitative Data Analysis (MAXQDA) version 18.^28^ The line-by-line coding of transcripts was based on operational definitions of key concepts of health literacy, including access, understand, appraise, and apply.^9^ Transcripts were not returned to participants because research has shown that interviewee transcript review (ITR) adds little to the accuracy of the transcript.^12^

### 2.6. Reflexivity

Determined efforts were made to fulfill the criteria of good qualitative research throughout the research process of data collection, data analysis, and reporting.^8,30,37^ Triangulation involving multiple researchers in the data collection and data analysis ensured internal validity. The external validity of this research was achieved by delineating the role of the researchers in the qualitative analysis and the description of the author contributions. Reliability and objectivity were accounted for in a reflective journal (based on field notes and reflective notes) summarized in an audit trail. During analysis, critical peer, but not participant feedback, sessions were held and searches for disconfirming evidence performed.

### 2.7. Ethics approval

The study was registered (W13_008 # 13.17.0021) with the Medical Ethics Committee of the Academic Medical Centre of Amsterdam, which declared that it did not fall under the scope of the “Medical Research Involving Human Subjects Act,” and all patients provided written informed consent before enrolment into the study. The study was reported using the consolidated criteria for reporting qualitative research.^35^

## 3. Results

### 3.1. Participants

Thirteen patients diagnosed with chronic musculoskeletal pain participated in this study: 4 men and 9 women aged from 21 to 77 years and with diverse educational levels (low, intermediate, and high). The participants differed in the levels of functional health literacy based on the raw scores of the SAHL-D. Based on the cutoff value of 54.5 of the SAHL-D, 1 of the 13 participants had adequate functional health literacy skills, and 12 were assessed as demonstrating low functional health literacy skills.^26^ The sociodemographic characteristics of the participants are summarized in Table 1. Twelve participants, 3 noncompleters (no noncompleter was highly educated) and 9 program completers, were interviewed 3 times during the pain management program. Contact was lost with 1 participant after the first interview (Jackie). This participant also withdrew from the IPMP, after a family member was diagnosed with a life-threatening illness.

**Table 1:** Participants' pseudonyms and sociodemographic characteristics.

Pseudonym	Gender	Age	Education level*	Health literacy level†	Health literacy level†	Status program completion
Rose	Female	57	Intermediate	54	Low	Noncompleter
Lia	Female	46	High	55	Adequate	Program completer
Ron	Male	37	Intermediate	46	Low	Program completer
Ann	Female	77	Intermediate	31	Low	Program completer
Bob	Male	66	Low	34	Low	Noncompleter
Jessy	Female	64	Intermediate	48	Low	Program completer
Kathleen	Female	76	Low	48	Low	Program completer
Jackie	Female	21	Low	22	Low	Noncompleter‡
Jim	Male	68	Intermediate	45	Low	Noncompleter
Sam	Male	44	Intermediate	51	Low	Program completer
Ellen	Female	31	Intermediate	43	Low	Program completer
Marissa	Female	35	High	50	Low	Program completer
Joan	Female	52	Intermediate	52	Low	Program completer

* Low (0–2: early childhood, primary education, lower secondary education), intermediate (3–5: upper secondary, postsecondary, short cycle tertiary), and high (6–8: bachelor, master's, doctoral).

† Health literacy level based on the SAHL-D.

‡ Noncompleter in the IPMP and research project after the first interview.

IPMP, interdisciplinary pain management programme; SAHL-D, Short Assessment of Health Literacy–Dutch.

### 3.2. Accessing information about pain before the interdisciplinary pain management programme

All participants described the ability to seek and obtain information on their condition before the IPMP. Some participants were very active in terms of seeking health information. Lia (46, adequate health literacy) described herself as a “critical” person. She reported how she preferred to solve her own problems and be well informed and well prepared when seeing a healthcare provider. She found it important to be convinced with arguments. Sam (44, low health literacy) described himself as a person who is capable of finding and comprehending relevant (scientific) information from the internet on his pain complaints. Different sources of health information were used by these “active” participants: internet (medical sites), magazines of patient organizations, medical leaflets, symposia, and conversations with healthcare providers.

Other participants, including Ellen (31, low health literacy), Ron (37, low health literacy), and Kathleen (76, low health literacy), relied on the expertise of their healthcare providers.

Ron described himself as a “doer,” who is busy all the time. He suffers from pain complaints since his car accident, which interfere with his work and family life. He was referred to the IPMP by his primary care physician because of his chronic pain complaints.

Kathleen (76, low health literacy) also described herself as a “doer”—and not that much of a “talker.” She was not critical and relied on the expertise of her doctor.

At the start of the IPMP, Bob (66, low health literacy) described himself as a person who was very active in seeking the right care for his pain problems. As a member of the patient spine organization, he tried to gain health information by reading the “Spine” magazine and attending symposia. He had a very defined idea about the cause of his pain.

### 3.3. Understanding information about pain neuroscience education

Most participants had difficulties recalling the information provided during the group PNE session: they were only able to describe fragments of the information and recalled some pictures they saw in the session.

Many referred to a picture of mountains (referring to the twin peaks model of the Explain Pain manual), but as Sam described, they were not able to recount the information on pain:

The only thing I remember is a picture of a mountain and mountaineers

Sam (44, low health literacy)

Most participants said that they did not understand the information. At the start of the program, Ron described how he did not know what the healthcare providers meant when they spoke about chronic pain; he had to look it up. He expressed how it is important that the information was interesting to him:

If it is not interesting to me, they can explain it 100 times, I won't receive the message

Ron (37, low health literacy)

Only a few participants were able to retell one of the important messages of the PNE session: the role of the brain in chronic pain, the concept of central sensitization.

However, they showed difficulty comprehending this information and seemed to not take this message seriously.

I think that my body, or maybe better said that my brain (ha ha, begins to laugh), my brain is trying to protect me

Marissa (35, low health literacy)

Lia and Sam were among the few participants, who said that they understood the health information. Sam described having learned from the interactions with his healthcare providers. He learned that his pain complaints were caused by an overactive pain system. He became conscious of his strategy of constant anticipation of pain stimuli, which prevented him from being physically active.

Lia described a conversation with her rehabilitation doctor at the IPMP intake, when she was told that her pain was caused by a chronic pain syndrome and was not based on the illness she thought she had. She understood the information and described how the doctor did not notice the impact of this message on her:

The chance was enormous that the pain I experience is not from mechanical impairments, but it is from the brain, the brain has a strong influence. I was shocked, flabbergasted at that moment

Lia (46, adequate health literacy)

### 3.4. Appraising information about pain

A few participants, including Lia, positively appraised the provided information. The new pain explanation was a real paradigm shift for Lia, with an enormous impact. She had to take a week off, she felt very stupid, and she realized that she had searched for help for many years in the wrong direction:

It is a bizarre revelation…with logical examples…that the penny has dropped. That your brain has such a bizarre influence on your pain experience… ehh yeah that was bizarre, you know… I learned this before… However, one way or another… I just never applied this to myself. Maybe my intelligence stood in the way to realize that I went that far in my self-care. Not to ask for help and just get on with it. A shame I struggled this long, but maybe it was smarter to ask for help sooner

Lia (46, adequate health literacy)

Many participants negatively appraised the information provided in the group PNE session. They described the information as too complex and boring. Jessy (64, low health literacy) said:

All that stuff with the brain, how it all works, that is difficult, you know, if you don't have the knowledge, I find it very difficult, I don't understand it all that fast, it's all so complicated

After the pain education session with the physiotherapist and the psychologist, Ron's appraisal was critical:

Pain is ouch, that's nothing for me! That doesn't help me

Ron (37, low health literacy)

He found it interesting to see the pictures of the brain and to learn how things work, but he described that it was of no use to him. He criticized the group format of the education session. He could not identify with the other participants, who equally suffered from pain complaints for many years. He was not interested in hearing the stories of the other participants and did not want to bother other participants with his complaints. He described how his attention waned during the education session. Ellen was also critical of the format of the education session:

It is useful of course, you always remember something, some small things, but 1.5 hours is just too long

Ellen (31 years old, low health literacy)

When Bob was asked to describe what he learned from PNE, he started to talk about a conversation he had with the rehabilitation team. In this conversation, the causes of Bob's pain complaints were discussed. He did not understand the information that was shared in this conversation:

I do not agree that it is caused here (he points to his head), I can hardly understand this. I can't believe this, maybe the doctor is right, but I look at it differently

Bob (66, low health literacy)

He described how he kept his own perspective on the cause of his pain. In this conversation with the rehabilitation team, it was decided that Bob had to stop with the IPMP because he did not agree with this information and was not able/willing to apply this information to his situation.

Participants described how they were distracted and had problems in concentrating. Sam (44, low health literacy) disliked the group format of the pain education session, finding the stimuli in the group too much. He expressed how he had not learned anything new and that he could not remember the information that was shared in this session:

For someone, with difficulties with concentration and focus… this should be done in a different way

Two participants reported that the pain education had a negative impact because their feelings were not taken seriously. Joan (52, low health literacy) described this as:

Chronic pain… they say it's in your head, that makes you think that it's all your own imagination the pain… but this is what I feel, I cannot walk because of the pain, it has consequences for how I function, that is not pain that I can describe as in my head… my imagination…

Kathleen (76, low health literacy) was critical of the pain education session:

It's all from the brain, that is of no use for me, I think I didn't understand it well. I thought, this isn't posturing, my complaints are real

Kathleen and Joan did not feel taken seriously as if they had exaggerated their complaints as a reaction to the message that pain is caused by the brain.

### 3.5. Applying information about pain

Almost all participants were unable to apply the information that was provided during the sessions in their daily life, describing it as of no use to them. Ron, for example, did not recognize how the pain education session related to his situation and was not able to apply it. Despite finding it difficult, a few were able to use the information. Lia, for instance, relied on her husband to reconstruct the health information. She reflected on her own self-care strategies and her old perspectives:

I realized that I had to change my mindset, I was “A” programmed and now I had to be “B” programmed. I tried to apply everything they told me, I tried to stay in the patient role, that was difficult for me, but in the end they told me that I was the ideal patient

Lia (46, adequate health literacy)

She learned to look at her pain in a different way. She expressed that she learned to differentiate between the mechanical and the mental influence on her pain. She learned to connect with her feelings and thoughts and how to relax and set limits.

Sam (44, low health literacy) learned that his pain complaints were caused by an overactive pain system. He became conscious of his strategy of constant anticipation of pain stimuli, which prevented him from being physically active. He tried to apply this knowledge and forced himself to become more physically active and not to focus on the pain itself:

I was used to focus on how to place my footsteps and not to run in a wrong way, now I try to distract myself to critically listen to my own music, to focus on something else than the running itself

Although overall Ron was pessimistic about applying the information, he could make use of the practical tips he got from the occupational therapy and social worker in his daily life:

The occupational therapist taught me to make my plan for the day, to think about whether it was not too much? From the social worker I learned to communicate with the people around me, when I'm too tired… It is all theory, but now I have to apply this

Ron (37, low health literacy)

## 4. Discussion

This qualitative study explored patient perspectives on information as part of PNE in an IPMP. All but one of the patients interviewed were assessed as having low health literacy (based on SAHL-D). As indicated by a recent study in Ireland, inadequate health literacy levels are common among IPMP patients.^12^ Yet, research on PNE has often overlooked the impact of low health literacy.^19,39^ Equally, in this study, PNE was not adapted to the level of health literacy of the participants by chunking information or using the teach-back method.^17^ Not surprisingly, many participants with low health literacy experienced difficulties in understanding and applying the PNE that was provided in the IPMP. In contrast with findings of a recent review of 4 qualitative studies, most participants showed no sign of reconceptualizing their pain (from a biomedical to a biopsychosocial understanding).^39^ This may have resulted from the conditions required for conceptual change not being met: (1) patients' ability to understand the concept, (2) the plausibility of the concept for patients, (3) patients' dissatisfaction with the current understanding of the pain, and (4) the practical usefulness of the concept in patients' everyday life.^27^ Health literacy levels likely played a role in this.^15,16,28,40^

Respondents (such as Kathleen and Bob) also had difficulties with the plausibility of the biopsychosocial understanding of pain, preferring their current understanding of pain. These findings echo those from an Irish study, which showed that low health literacy was related with lower disease-related knowledge about pain.^20^ By contrast, one participant with adequate health literacy acknowledged the plausibility of the concept, reporting how she saw the value of changing her mindset.

Several participants reported problems with attention and memory: problems that have been acknowledged in other research on chronic pain and depression.^4,31^ The group format of the PNE may have complicated information processing for these participants and contributed to these problems. In addition, the total time spent on education may not have been enough, although the dose–response relationship of PNE is uncertain. A recent study showed that educationally, cognitively, and literacy-disadvantaged patients seemed to benefit more from a literacy-adapted cognitive-behavioural treatment (CBT) than from education. The directive, skills-building approach of CBT might have increased the benefit for patients with cognitive or educational barriers by providing more necessary guidance and support for how to apply the information in their lives outside of the treatment.^7^ This fits the more practical learning style of Kathleen and Ron (describing themselves as “doers”). Other participants with a more cognitive learning style (finding scientific information on the internet: Lia and Sam) may benefit more not only from group education and group discussions but also from handouts, flyers, and/or videos. The findings suggest that the group format did not sufficiently address participants' individual needs. Other researchers have shown the limited efficacy of group PNE for patients with low health literacy.^7^ Tailoring health information to patients with low health literacy has showed small improvements for knowledge in musculoskeletal interventions.^19^ An individualized, more concrete, and structured pain education program may be needed for literacy-disadvantaged patients. Further research is needed to investigate whether such a structured program benefits literacy-disadvantaged patients with chronic pain. More research is also needed on which dosage and format of PNE delivery is most effective in reconceptualizing pain in all patients with chronic pain, but especially those with low health literacy.

## 5. Strengths and limitations

Considerable measures were undertaken to improve the validity of the study findings (triangulation involving multiple researchers, promoting reflexivity during the whole research process) and reliability and objectivity (following an iterative process of induction and deduction, during the analyses, constant comparison within and between participants was applied, and sceptical peer-review sessions were organized to facilitate the search for disconfirming evidence during the analysis).^8,30,35,37^

Although the health literacy levels of the participants were assessed with a valid objective measurement, the SAHL-D,^26^ the interactive and critical health literacy dimensions of the Integrated Conceptual Model of Health Literacy were not assessed.^32^

The findings are limited by the absence of participants with very low health literacy (1 dropped out before the follow-up interview) and the low number (1) with high health literacy levels. However, the level of health literacy aligns with patients attending interdisciplinary pain management programs elsewhere.^20^

## 6. Implications of research and practice

Tailoring the information in PNE with consideration for patients' health literacy levels, information needs, and learning strategies is important. Lessons can be learned from the extensive literature on health literacy and patient education: decrease the complexity of the information by using plain language; be specific and limit the information (“askme3” questions: (1) what their main problem is, (2) what they need to do, and (3) why it is important to them), and make sure that a patient has understood the information given (apply the teach-back method).^6,11,13,34,38,42^

## 7. Conclusions

This is the first qualitative study to explore patient perspectives on PNE in an IPMP. Most participants did not seem to receive the health information in PNE as intended and experienced difficulties with understanding the message, negatively appraised the information, and were not able to apply this in their everyday lives.

## Disclosures

The authors have no conflict of interest to declare.

## Appendix A. Supplemental digital content

Supplemental digital content associated with this article can be found online at http://links.lww.com/PR9/A203.
